# Bent DNA Bows as Sensing Amplifiers for Detecting DNA-Interacting Salts and Molecules

**DOI:** 10.3390/s20113112

**Published:** 2020-05-31

**Authors:** Jack Freeland, Lihua Zhang, Shih-Ting Wang, Mason Ruiz, Yong Wang

**Affiliations:** 1 Department of Physics, University of Arkansas, Fayetteville, AR 72701, USA; jfreelan@uark.edu (J.F.); mtruiz@uark.edu (M.R.); 2 Department of Chemistry and Biochemistry, University of Arkansas, Fayetteville, AR 72701, USA; 3 Center for Functional Nanomaterials, Brookhaven National Laboratory, Upton, NY 11973, USA; lhzhang@bnl.gov (L.Z.); shihwang@bnl.gov (S.-T.W.); 4 Department of Biology, University of Arkansas, Fayetteville, AR 72701, USA; 5 Cell and Molecular Biology Program, University of Arkansas, Fayetteville, AR 72701, USA; 6 Microelectronics-Photonics Program, University of Arkansas, Fayetteville, AR 72701, USA

**Keywords:** nucleic acids, interactions, elasticity, bending, molecular spring

## Abstract

Due to the central role of DNA, its interactions with inorganic salts and small organic molecules are important. For example, such interactions play important roles in various fundamental cellular processes in living systems and are involved in many DNA-damage related diseases. Strategies to improve the sensitivity of existing techniques for studying DNA interactions with other molecules would be appreciated in situations where the interactions are too weak. Here we report our development and demonstration of bent DNA bows for amplifying, sensing, and detecting the interactions of 14 inorganic salts and small organic molecules with DNA. With the bent DNA bows, these interactions were easily visualized and quantified in gel electrophoresis, which were difficult to measure without bending. In addition, the strength of the interactions of DNA with the various salts/molecules were quantified using the modified Hill equation. This work highlights the amplification effects of the bending elastic energy stored in the DNA bows and the potential use of the DNA bows for quantitatively measuring DNA interactions with small molecules as simple economic methods; it may also pave the way for exploiting the bent DNA bows for other applications such as screening DNA-interacting molecules and drugs.

## 1. Introduction

DNA is one of the most essential elements of life, and its interactions with inorganic salts and small organic molecules are important for many reasons [1,2]. First, understanding these interactions is critical for understanding various fundamental cellular processes in living systems [1,3,4,5]. For example, genomic stability and DNA repair rely on the presence, mediation, and/or participation of metal ions [1,6,7,8]. Second, the DNA interactions with salts/molecules are important for the mechanism of diseases, especially those related to DNA damaging and repairing [9,10,11]. For example, heavy metal ions and various chemical carcinogens and mutagens interact and react with DNA directly or indirectly, causing many human cancers [12,13,14]. Third, understanding these DNA interactions helps to discover, design and develop inhibitors and drugs targeting DNA for treating various diseases [15,16,17]. For example, DNA damaging agents have been widely used in treating many cancers [16,17,18]. Therefore, it is important to understand the interactions between DNA and inorganic salts or small organic molecules.

Various techniques have been developed for investigating the interactions between DNA and other molecules, including gel electrophoresis, melting-curve, fluorescence anisotropy, circular dichroism, isothermal titration calorimetry, x-ray absorption spectroscopy, optical tweezers, magnetic tweezers, electron paramagnetic resonance, Raman spectroscopy, and nuclear magnetic resonance spectroscopy [19,20,21,22,23,24,25,26,27,28,29,30,31,32,33,34,35,36,37]. However, strategies that improve the sensitivity of the existing techniques and methods would be appreciated because they help to identify molecules that interact with DNA too weakly and to make new discoveries [38]. In addition, when expensive, sophisticated equipment is not available, the need to develop such a general strategy is more pressing [38].

**Figure 1:**
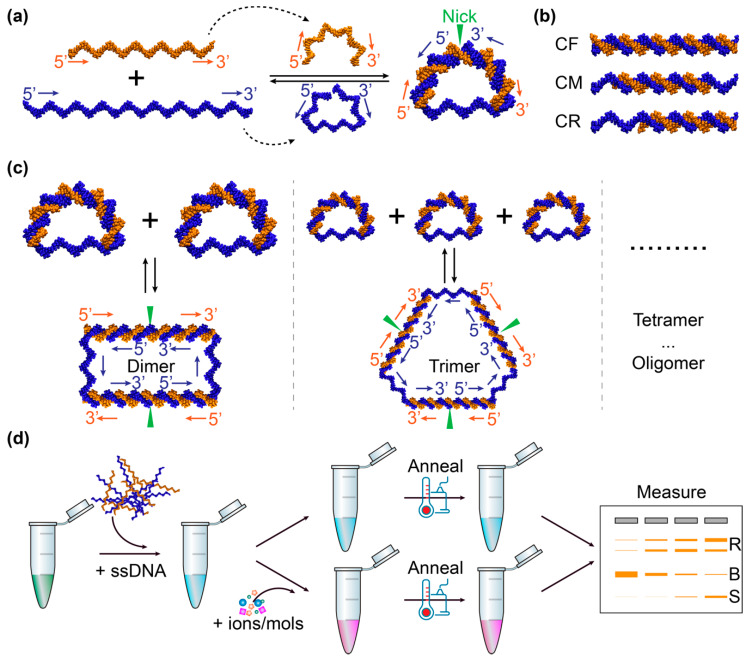
Illustration of sensing amplifiers based on bent DNA bows. (**a**) Construction of DNA bows from synthesized single-stranded DNA. The 5′- and 3′- ends of the DNA strands are indicated, along with arrows from 5′ to 3′. The nick in the bent DNA bow is highlighted by the green triangle. (**b**) Linear unbent DNA as negative controls. CF: fully double-stranded DNA; CM: partially double-stranded DNA in the middle; CR: partially double-stranded DNA on the right. (**c**) Relaxation of bending elastic energy in DNA bows by forming dimers, timers, and higher-order oligomers. The 5′- and 3′ ends of the DNA strands, the direction from 5′ to 3′, and the location of the nicks are highlighted similarly to panel a. (**d**) Sketch of the procedure for detecting DNA interactions with ions and molecules, visualized by gel electrophoresis as an example. R stands for the bands corresponding to the relaxed species (dimers, trimers, etc.). B indicates the band for the bent DNA bows. S represents the band for the unhybridized single-stranded DNA.

In this work, we demonstrated the application of bent DNA bows [39,40,41,42] as sensing amplifiers for detecting and measuring the interactions of DNA with 14 different inorganic salts and small organic molecules. The DNA bows were constructed as illustrated in Figure 1a, following the previous work [39,40,41,42]. Briefly, two single-stranded DNA sequences are designed such that the left 1/3 of the long sequence (blue) hybridizes to the left half of the short sequence (orange), while the right 1/3 of the long sequence hybridizes to the right half of the short sequence, leaving the middle 1/3 of the long sequence unhybridized (Figure 1a) [39,40,41,42]. Therefore, it is possible to produce, upon hybridization, a looped DNA molecule that consists of two segments: a double-stranded segment with a nick and a single-stranded part (Figure 1a) [39,40,41,42]. If the contour length of the double-stranded segment is longer than that of the single-stranded part, the double-stranded segment is bent while the single-stranded part is stretched – like an archery bow. The resultant mechanical energy due to the bending and stretching of the DNA bows puts them in energetically unfavored states, and makes them more susceptible and sensitive, compared to linear unbent DNA molecules (Figure 1b), to perturbations caused by the interactions of DNA with other molecules [41,43,44]. For example, the bending elastic energy in the DNA bows helps to drive the formation of dimers, trimers, and higher-order oligomers (Figure 1c) or to stimulate the dissociation of double strands into single strands, as the elastic energy gets released in the dimers (and trimers, tetramers, etc.) or the single strands [39,40,41,42]. Due to the enhanced susceptibility, the bent DNA bows enabled us to detect these interactions much more easily than unbent DNA, and thus to improve the sensitivity of the existing methods and techniques, among which gel electrophoresis was used as an example in this work. In addition, we showed that this technique based on bent DNA bows was capable of quantifying these interactions by fitting the relationship between the percentage of bent DNA bows and the concentration of the tested salts or molecules in the solutions using the modified Hill equations, which report the strength of the DNA-salt or DNA-organic compound interactions.

## 2. Materials and Methods

### 2.1. Construction of DNA Bows

Synthetic DNA oligos were purchased from Integrated DNA Technologies (Coralville, IA, USA), purified by standard desalting by the vendor, and resuspended in distilled water to a final concentration of 100 µM. The oligos used in this study include S45 (5′ - CAC AGA ATT CAG CAG CAG GCA ATG ACA GTA GAC ATA CGA CGA CTC -3′), S30B (5′ - CTG CTG AAT TCT GTG GAG TCG TCG TAT GTC - 3′), S45CF (5′ - GAG TCG TCG TAT GTC TAC TGT CAT TGC CTG CTG CTG AAT TCT GTG - 3′), S30CM (5′ - GTA TGT CTA CTG TCA TTG CCT GCT GCT GAA - 3′), and S30CR (5′ - TAC TGT CAT TGC CTG CTG CTG AAT TCT GTG - 3′).

Bent DNA bows were constructed from two synthesized single-stranded DNA (S45 and S30B) via self-assembly following our previous work [41]. The sequences of the single strands were designed such that the last 15 nucleotides at the 5′-end of the long strand (S45) hybridized to the 5′-half of the short strand (S30B), while the last 15 bases at the 3′-end of S45 hybridized to the 3′-half of S30B. Upon hybridization, a circular construct was formed, with a double-stranded segment of 30 base pairs (bp) with a nick and a single-stranded segment of 15 bases [39,40,41,42]. Three linear constructs (CF, CM, and CR, shown in Figure 1b) were used as negative controls. Upon hybridization, CF (S45 + S45CF) were double stranded completely, while CM (S45 + S30CM) and CR (S45 + S30CR) had overhangs of single strands at one or two sides, respectively. The long strands for CF and CM were the same as the long one in the DNA bows.

### 2.2. Detection of DNA-Interacting Salts/Molecules Using DNA Bows

The detection of DNA-interacting salts/molecules using DNA bows is illustrated in Figure 1d. Briefly, single strands (S45 and S30B) were mixed at equal molar amounts in background buffer (0.4 mM Tris-HCl with pH adjusted to 7.5 and 0.5 mM NaCl) to reach a final concentration of 2 µM [41]. It is noted that the concentrations of NaCl and Tris in the background buffer were much lower than those in commonly used hybridization buffers, such as the SSC buffer, in order to achieve better performance of the bent DNA bows. The DNA samples without other ions or molecules were used as baselines/controls. To detect possible interactions of ions and molecules of interest, solutions of the salts/molecules of interest were prepared in water and mixed with DNA strands in the background buffer to reach desired concentrations (Table 1). The ranges of concentrations were selected so that clear changes could be observed for the band patterns in gel electrophoresis. The mixtures were heated to 75 °C for 2 min and gradually cooled down to 22 °C (room temperature) in 5 hr [41]. The mixtures were incubated at 22 °C for overnight to allow full equilibrium, followed by gel electrophoresis for visualization on the second day [41].

### 2.3. Gel Electrophoresis

Polyacrylamide gels (12%) were prepared in the laboratory. Briefly, 3 mL of 40% acrylamide/bis solution 19:1 (Bio-Rad Laboratories, Hercules, CA, USA), 1 mL of 10X tris-borate-EDTA (TBE) buffer (Bio-Rad Laboratories), 20 µL of freshly made ammonium persulfate (APS, 10% in water, Thermo Fisher Scientific, Waltham, MA, USA) and 6 mL of distilled water were mixed thoroughly and degassed for 5 min in vacuum. The mixture was poured into gel cast cassette immediately after adding 10 µL of tetramethylethylenediamine (TEMED, Thermo Fisher Scientific), followed by incubation at room temperature for 20–60 min to allow full polymerization before use.

The prepared DNA samples (5 µL) were mixed thoroughly with 1 µL of 6X DNA loading buffer (Bio-Rad Laboratories). The mixtures were loaded into the wells of the prepared gel. The gel electrophoresis (Edvotek Inc., Washington, DC, USA) was run at 100 V for 50–60 min in 1X TBE buffer, followed by staining the gel with 1X SYBR Safe solution (Thermo Fisher Scientific) for 15–30 min with gentle shaking. The stained gel was then imaged with a typical exposure time of 1–5 s using a gel documentation system (Analytik Jena US LLC, Upland, CA, USA).

The acquired gel images were analyzed using ImageJ [46,47]. The original gel images were first rotated, cropped, and inverted, followed by subtracting the background with a rolling ball radius of 10 pixels [48]. The preprocessed gel lanes were then analyzed using the gel analysis procedure in ImageJ [46,47], from which the intensities of the bands of DNA bows and that of the bands corresponding to the relaxed ones (dimers, trimers, and oligomers) were obtained. Lastly, the intensities were rescaled/normalized by dividing the original values of the band intensities by the band intensity of the DNA bows (i.e., the band indicated by B in Figure 1d; or the double-stranded band for the negative controls) on the same gel in the absence of ions/molecules of interest (i.e., the 0 mM or 0 µM lanes) [41].

### 2.4. Visualization of DNA Bows Using Transmission Electron Microscopy (TEM)

The morphology of the bent DNA bows was visualized by TEM imaging. Briefly, the DNA bows (~2 µM, prepared in the background buffer with 0.4 mM Tris-HCl at pH 7.5 and 0.5 mM NaCl) were diluted in the background buffer to a final concentration of ~200 nM. Then, 5 μL of the diluted DNA solution was dropped onto a carbon film coated TEM grid (Electron Microscopy Sciences, Hatfield, PA, USA), and incubated at room temperature for 1.5 min, followed by removing the residual liquid with filter papers. The grid was washed by 5 μL of deionized water and stained with 5 μL of 2 wt% Nano-W^TM^ (Nanoprobes Inc., Yaphank, NY, USA) for 10 s [49]. After removing excess liquid with filter papers, the DNA sample was imaged using a JEOL 2100F TEM with an acceleration voltage of 200 kV.

## 3. Results

### 3.1. Visualization of Bent DNA Bows Using TEM

**Figure 2:**
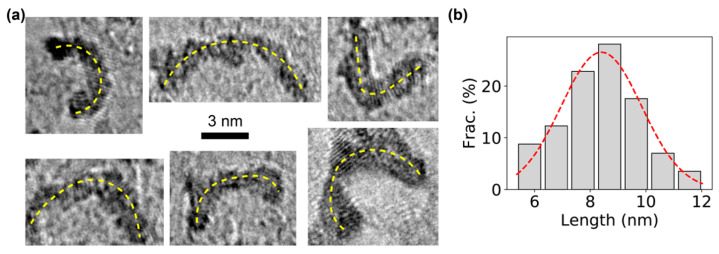
TEM visualization of bent DNA bows. (**a**) Examples of TEM images of DNA bows. The bending of the DNA bows is highlighted by the yellow dashed lines. Scale bar = 3 nm. (**b**) Distribution of the arc lengths of DNA bows, which was fitted by a Gaussian distribution (red dashed line), resulting in an average length of 8.4 ± 1.4 nm (mean ± standard deviation).

TEM imaging was performed to directly visualize the constructed DNA bows after negative staining with organo-tungstate compounds (Nano-W^TM^) [49]. Examples of TEM images of individual DNA bows clearly showed the bending structures (Figure 2a), which are presumably the bent, double-stranded part of the DNA bows. For confirmation, the arc lengths of the bending structures on the TEM images were quantified using ImageJ [46,47]. A single peak was observed in the distribution of the measured arc lengths (Figure 2b). Fitting the peak with the Gaussian distribution resulted in an average length of 8.4 ± 1.4 nm (mean ± standard deviation). Considering that the double-stranded segment of DNA bows has a length of 30 bp, the measured arc length of the DNA bows was consistent with previous reports from direct TEM imaging of DNA structures and X-ray data [50], confirming that the dark bending structures in the TEM images were indeed bent, double-stranded segment of the DNA bows.

### 3.2. Detection of Inorganic Ions Using Bent DNA Bows

The DNA bows were applied to detect the interactions of DNA with various inorganic ions. It is well-known that these DNA-ion interactions play essential roles in the properties and functions of DNA molecules [1,8]. One of the most famous examples is the local and long-range electrostatic interactions of cations on the structure and stiffness of DNA [19,21,51]. In particular, Mg^2+^ ions are well-known to mediate and stabilize the secondary structures of DNA, playing important roles in genomic packaging, gene regulation, and DNA-repairing [19,21,51,52]. On the other hand, certain ions, especially heavy metal ions such as Al^3+^, Ag^+^, and Zn^2+^, can damage DNA molecules [53,54,55,56,57], accumulation of which is associated with various diseases including cancers [12,13,14].

**Figure 3:**
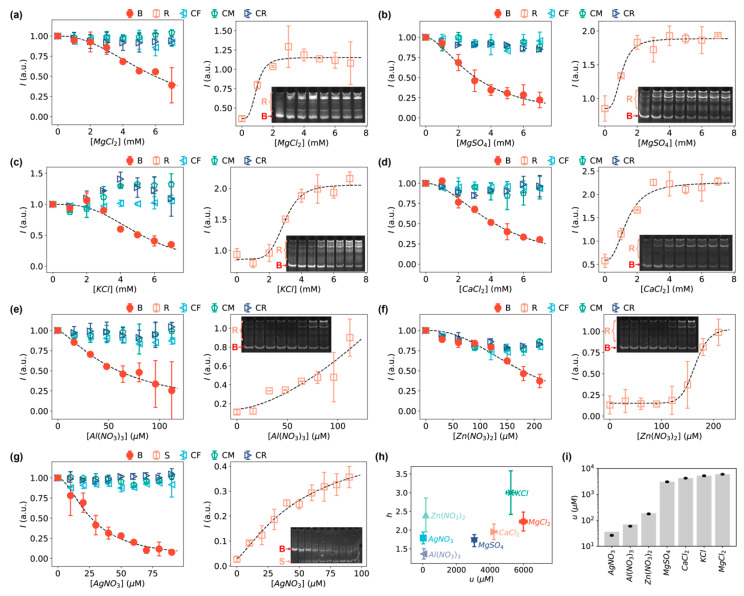
Intensities of the bands of DNA bows (red circles), relaxed DNA loops (orange squares), and linear DNA controls (cyan triangles, green pentagons, blue triangles) in the presence of various salts at increasing concentrations: (**a**) MgCl_2_, (**b**) MgSO_4_, (**c**) KCl, (**d**) CaCl_2_, (**e**) Al(NO_3_)_3_, (**f**) Zn(NO_3_)_2_ and (**g**) AgNO_3_ (the orange squares for AgNO_3_ are for the dissociated single strands S). Insets are the representative, cropped gels of bent DNA bows in the presence of the corresponding salts at increasing concentrations. B and R (or S) indicate the bands used for quantification of the intensities. The corresponding full-length gels are shown in Figure S1. Error bars in panels a–g represent the standard deviation from 2–4 replicates. (**h**) Fitted h and u values from the modified Hill equation for the inorganic salts. (**i**) Fitted u-values for quantifying the strength of DNA interactions with inorganic salts.

We first examined the interaction of DNA with Mg^2+^ ions from two different salts, MgCl_2_ and MgSO_4_, and found that both salts were capable of driving the formation of relaxed DNA loops (Figure 3a,b). Without the bent DNA bows to amplify the signals (i.e., with linear DNA controls as shown in Figure 1b), the DNA molecules treated with MgCl_2_ or MgSO_4_ at concentrations up to 7 mM did not show any observable difference in gel electrophoresis (Figure S1, gels indicated by “CF”, “CM”, and “CR”). Note that this observation also indicated that the migration of the DNA was not affected by the salts at these concentrations. Quantifying the intensities of the bands showed little difference for the increasing concentrations of Mg^2+^ ions (◁, ▷, and ⬠ in Figure 3a,b). Interestingly, when amplifying the signals of the DNA interactions with the MgCl_2_ and MgSO_4_ salts using the bent DNA bows, changes in the gel electrophoretic patterns of the DNA molecules were clear (Figure 3a,b, and gels indicated by “Bent” in Figure S1). The intensities of the DNA bows decreased as the concentrations of the Mg^2+^ salts increased (● in Figure 3a,b). In addition, heavier bands corresponding to relaxed DNA loops (such as dimers, trimers, and/or oligomers) appeared in the presence of Mg^2+^ salts (insets of Figure 3a,b) [41]. By quantifying the intensities of the relaxed species (i.e., all other bands except the bands of DNA bows), we confirmed that the intensities of relaxed DNA loops increased, reaching a plateau at Mg^2+^ concentration ≈ 3 mM (□ in Figure 3a,b).

In addition to the Mg^2+^-salts, we tested K^+^-salt (KCl) and Ca^2+^-salt (CaCl_2_) that benefit various cellular processes [58,59,60,61,62], and three other ions (Al^3+^, Zn^2+^, and Ag^+^, provided from the corresponding nitrate salts) that have been shown to be closely related to DNA damage *in vivo* [15,16,17,53,54,55,63,64]. The K^+^ and Ca^2+^ ions resulted in similar effects on the DNA molecules in the same range of salt concentrations from 0 to 7 mM (Figure 3c,d, and Figure S1): the band intensities of the DNA bows decreased while the intensities of the relaxed, heavier species increased. For the DNA-damaging ions, we observed that both Al^3+^ and Zn^2+^ ions resulted in the formation of heavier relaxed DNA loops (Figure 3e,f), similar to Mg^2+^ ions; however, Ag^+^ ions led to dissociation of the bent DNA bows (Figure 3g), as the band corresponding to the single-stranded DNA appeared [41]. Quantifying the intensities of the single-stranded bands (S) showed clear increases (orange squares in Figure 3g) [41]. The difference in the change of gel patterns caused by the ions between Ag^+^ and all the other tested ions suggests that their interactions with DNA are distinct. Also note that the working concentrations of Al^3+^, Zn^2+^ and Ag^+^ ions were 10–100 times lower than that of Mg^2+^, K^+^, and Ca^2+^ ions.

To quantify the strength of the DNA-salt interactions by the bent DNA bows, we fitted the normalized band intensities of the DNA bows $I_{B}$ as functions of the concentrations of the salts using an equation derived from the Hill equation that has been extensively used for characterizing the binding between ligands and macromolecules [65,66],

(1) $$I_{B}=1-\frac{c^{h}}{c^{h}+u^{h}}=\frac{u^{h}}{c^{h}+u^{h}}$$

where c is the concentration of the tested salts, h is the Hill coefficient, and u is the characteristic concentration of the tested salts producing half of the band intensity of the DNA bows in the absence of the salts. It turns out that equation (1) fitted all the data very well (Figure 3). The fitted parameters (h and u) were presented in Figure 3h for the seven tested inorganic salts. In addition, the characteristic concentrations (u) for different salts were compared in Figure 3i, which suggested that Al(NO_3_)_3_, Zn(NO_3_)_2_ and AgNO_3_ showed stronger interactions with DNA (i.e., lower u values) compared to MgCl_2_, MgSO_4_, KCl, and CaCl_2_.

In addition to the bands of DNA bows, we quantified the summed intensities of the relaxed bands $I_{R}$ and fitted them using the Hill equation [65,66] with the addition of a baseline (b),

(2) $$I_{R}=I_{max}\times \frac{c^{h^{\prime }}}{c^{h^{\prime }}+{u^{\prime }}^{h^{\prime }}}+b$$

where $I_{max}$ is the maximum intensity of the relaxed bands measured from the baseline. As shown in Figure 3, the data from the relaxed bands can also be fitted well with the modified Hill equation, providing an additional way to quantify the interactions of the inorganic salts with DNA. Note that the two parameters in the Hill equation ($u^{\prime }$ and $h^{\prime }$) obtained from the relaxed bands are not necessarily the same as those estimated from the bands of DNA bows ($I_{B}$), because the relaxed bands contain multiple relaxed species of different orders (i.e., dimers, trimers, tetramers, and higher-order oligomers). Nonetheless, we found that $u^{\prime }$ correlated very well with u for all the tested inorganic salts, except for Al(NO_3_)_3_ (Figure S3). We also point that the fitted parameters from $I_{R}$ are expected to be less reliable than those from $I_{B}$ for several reasons. First, the R bands were smeared much more significantly than the B bands and thus less well-defined. More importantly, as background subtraction was performed in order to minimize human bias in the quantification of the band intensities, certain smears of the R bands were removed during this process, leading to inaccuracy in the intensity quantification of the R bands. In addition, we noticed that the smearing varied slightly among different batches of synthesized DNA oligos and among different gels, which introduced additional uncertainty in the quantified intensities of the R bands. These reasons led to larger variations and higher uncertainties in $I_{R}$. Therefore, we suggest that the bent DNA bands are more reliable for quantitative analysis. Nonetheless, the R bands provide a way for cross validation.

### 3.3. Detection of Small Organic Molecules Using Bent DNA Bows

In addition to inorganic salts, we applied the bent DNA bows to amplify and detect the interactions of DNA with small organic molecules (Table 1). First, guanidine (guanidinium chloride, or guanidine hydrochloride, GuHCl) were tested as it is a commonly used chaotropic agent at high concentrations to denature double-stranded DNA [67,68]. Second, salts of putrescine and spermidine were chosen for testing our DNA bows because they have been reported previously to interact with DNA and shorten the persistence length of DNA [21,69,70]. Third, we tested EtBr and SYBR safe, which are commonly used DNA intercalators and DNA staining dyes in gel electrophoresis [71,72,73]. Lastly, we tested two molecules that are relevant to the production of DNA but unknown direct interactions with DNA: ganciclovir and thiamine. Ganciclovir is an antiviral medication used to treat cytomegalovirus (CMV) infections, and ganciclovir triphosphate is a competitive inhibitor of deoxyguanosine triphosphate (dGTP) incorporation into DNA [74,75]. Thiamine is a vitamin that serves as a cofactor for a series of enzymes in different metabolic pathways and is required for the production of ATP, ribose, NAD, and DNA [76,77]. It was reported that derivatives of thiamine bind to messenger RNAs and regulate gene expression in bacteria [78]; however, to our knowledge, direct interaction between thiamine and DNA has never been observed.

**Figure 4:**
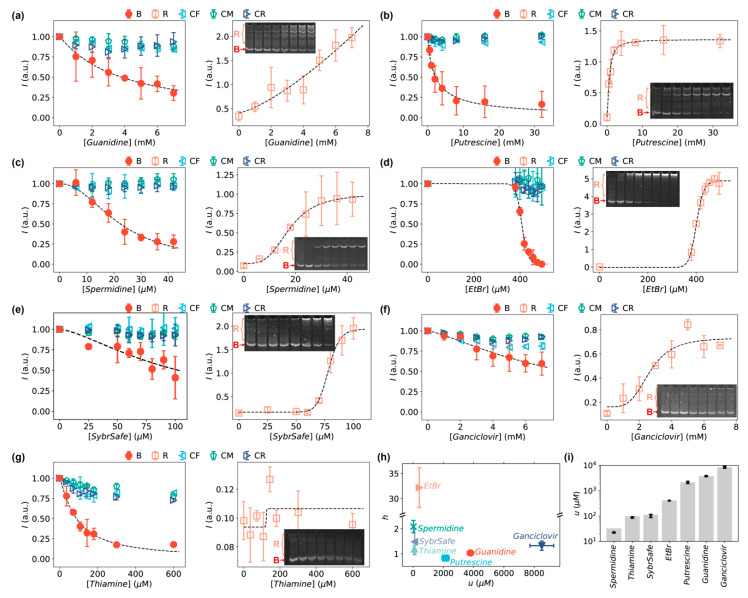
Intensities of the bands of DNA bows (red circles), relaxed DNA loops (orange squares), and linear DNA controls (cyan triangles, green pentagons, blue triangles) in the presence of small organic molecules at increasing concentrations: (**a**) guanidine, (**b**) putrescine, (**c**) spermidine, (**d**) ethidium bromide, (**e**) SYBR safe, (**f**) ganciclovir, and (**g**) thiamine. Insets are the representative, cropped gels of bent DNA bows in the presence of the corresponding small organic molecules at increasing concentrations. The corresponding full-length gels are shown in Figure S2. Error bars in panels a–g represent the standard deviation from 2–4 replicates. (**h**) Fitted h and u values from the modified Hill equation for the organic molecules. (**i**) Fitted u-values for quantifying the strength of DNA interactions with organic molecules.

As expected, the interactions between DNA and guanidine, putrescine, spermidine, EtBr, or SYBR Safe could be amplified and detected by the bent DNA bows. It is noted that these interactions were not detectable or not significant without amplification by the bent DNA bows (i.e., with linear double-stranded DNA controls) at low enough concentrations of these organic molecules (gels indicated by “CF”, “CM”, and “CR” in Figure S2). In contrast, when amplifying the signal of the DNA interactions with these organic molecules using the bent DNA molecules, the effects of the molecules at the same concentrations were observed (Figure 4a–e, and gels indicated by “Bent” in Figure S2). Similar to the inorganic salts, the intensities of the bent DNA bands decreased as the concentrations of the organic molecules increased; however, the ranges of the “working” concentrations of the organic molecules were more diverse than the inorganic salts. This diversity suggests that the observed interactions of organic molecules with the DNA bows were unlikely due to the residue ions in the solutions of organic molecules. In addition, the appearance of the heavier bands suggested that guanidine, putrescine, spermidine, EtBr, and SYBR Safe caused the formation of relaxed dimers or oligomers (Figure 4a–e). It is worthwhile to point out that the patterns of the heavier bands are different for different organic molecules, and some of the patterns are distinct from that of the inorganic salts (e.g., Figure 4c–e), which again suggests that the interactions of DNA with different organic molecules and inorganic salts are different.

Interestingly, we observed that our bent DNA bows were also able to amplify and detect the interactions of DNA with ganciclovir and thiamine. For ganciclovir, the intensities of the bent DNA bands decreased, while faint bands of heavier species emerged as the concentration of ganciclovir increased (Figure 4f). In contrast, heavier bands were absent as the concentration of thiamine increased, even if the intensities of DNA bows decreased (Figure 4g). The decrease cannot be completely attributed to the interference of thiamine with DNA staining using SYBR safe after running the gel, as the decrease in the linear double-stranded DNA controls was weaker (Figure 4g and Figure S2).

Similar to the inorganic salts, the strength of the DNA interactions with these organic molecules were quantified using the modified Hill equations (equation (1) for $I_{B}$, and equation (2) for $I_{R}$), which again fitted all the data very well (except for thiamine as there were not relaxed bands), as shown in Figure 4. The fitted parameters from $I_{B}$ (h and u) were presented in Figure 4h for the seven tested organic molecules. In addition, the characteristic concentrations (u) for different organic molecules were compared in Figure 4i. Furthermore, a good correlation between $u^{\prime }$ obtained from $I_{R}$ and equation (2) correlated and u was observed for all the organic molecules (except guanidine), although the fitting error of $u^{\prime }$ for thiamine was large (Figure S3), although we expect that the bent DNA bands are more reliable for quantitative analysis for the same reasons as we described for the inorganic salts.

## 4. Discussions and Conclusions

In conclusion, we demonstrated the application of bent DNA bows as amplifiers and sensors for detecting and quantifying the interactions between DNA and 14 different inorganic salts and small organic molecules. These interactions were difficult to detect and visualize using gel electrophoresis with unbent DNA strands; however, our bent DNA bows were able to amplify these interactions, making them much easier to visualize and quantify. The amplification was facilitated by the bending energy in the bent DNA bows, which drove the conversion of the bent DNA bows to relaxed species, such as relaxed loops (i.e., straightened double-stranded segment) or dissociated single-strands [41,79]. In addition, this technique based on bent DNA bows were capable of quantify the DNA interactions with the various inorganic salts and small organic molecules by fitting the relation between the amount of bent DNA bows and the concentrations of the tested salts or molecules presented in the solutions (i.e., $I_{B}$ vs. c) using the modified Hill equations (Equations (1) and (2)). The strength of the interactions of the tested salts and molecules with DNA can be reported by the characteristic concentration u in the modified Hill equation.

We would like to highlight the novelty of the current work, which is a reduction to practice of the concept of using bent DNA bows as sensing amplifiers, which we reported previously [41]. First, the concept was rigorously tested and validated by 12 additional inorganic salts and organic molecules in this work. More importantly, the application of the bent DNA bows to the study of DNA interactions with organic molecules has never been reported previously. In addition, the bent DNA bows allowed us to observe the direct interaction between thiamine and DNA for the first time.

Instead of the commonly used concepts for sensors (e.g., sensitivity, limit of detection, and dynamic range), we have chosen to use the μ and h parameters from the Hill equations to characterize the bent DNA bows as sensing amplifiers. One reason of this choice is that the sensitivity of biosensors, typically defined as the ratio of the change in signal to the change in analyte concentration, is only useful within the linear range [80], while the measured concentration dependences were sigmoid and deviated obviously from linearity (Figure 3 and Figure 4). Another reason is that the limit of detection and dynamic range can be derived from the parameters of the Hill equations. Therefore, in our opinion, the parameters of the Hill equations are more fundamental and more relevant here.

The Hill equations was used to fit experimental data (Figure 3 and Figure 4) because the conversions between the bent DNA bows and the relaxed species (i.e., dimers, trimers, and higher-order oligomers) could be considered as biochemical “reactions”. The success of the fittings supported the validity of this concept. The fitted Hill equation parameters (μ and h) could be potentially used for estimating various parameters of the interactions, such as the association/dissociation constants. In addition, the difference in the Hill coefficient (h) observed for different ions/molecules could possibly have important implications on their interactions with DNA. On the other hand, it is worthwhile to note that the fitted h parameter is combination of individual Hill coefficients from the individual “reactions” for the formation of dimers, trimers and higher-order oligomers. It is difficult to decompose the fitted h parameters in the current study; however, analytical high-performance liquid chromatography or mass spectroscopy are likely able to distinguish and identify the individual relaxed species and thus facilitate a deeper understanding of the DNA interactions with the ions/molecules from the fitted h parameters.

This work highlights the amplification effects of bent DNA bows on the interactions between DNA and other molecules. The demonstrations using 14 inorganic salts and organic molecules tested in this study suggested that the bent DNA bows could serve reliably as sensing amplifiers for many DNA-interacting molecules. We also point out that the bent DNA bows are limited in distinguishing different ions or molecules. In other words, although the strengths of the interactions of different inorganic salts and organic molecules with the bent DNA bows are different, it is expected to be practically difficult to reliably trace back to the type of ions/molecules solely based on the strengths (i.e., u values or $I_{B}$-c curves). On the other hand, we would like to emphasize that such limitation does not prevent the bent DNA bows from being good sensing amplifiers in certain applications. Just like a light sensor that does not distinguish colors could be useful in applications where uniform responses to a wide spectrum are desired (e.g., monochromatic CCD cameras in fluorescence microscopy), the bent DNA bows could be great sensing amplifiers for screening a pool of molecules and identifying the candidates that interact with DNA in pharmaceutical applications [81,82].

The key fundamental idea behind the concept of using bent DNA bows as sensing amplifiers is that the elastic energy introduced by the bending of the double-stranded DNA segments makes them more susceptible to perturbations caused by salt ions and organic molecules, and thus amplifies the signal of the interaction between the DNA and the salts/molecules. It is worthwhile to point out that there are several different strategies to introduce bending in double-stranded DNA. The bent DNA bows described in this work is one of them. Another strategy uses a double-stranded segment in the middle with two sticky single strands on both ends, which forms a loop upon hybridization of the sticky ends. This strategy has been extensively used in cyclization experiments [83,84,85,86,87,88,89,90,91,92,93], which have significantly advanced our understanding on the flexibility of DNA. However, a potential shortcoming of this strategy lies in the practical difficulty to achieve extremely short loops (<80 bp) as shorter loops are energetically unfavored [83,84,85,86,87,88,89,90,91,92,93]. A third strategy to achieve bent double-stranded DNA is through a hairpin structure, introduced by the Cohen group [94]. We expect that the other two strategies would be suitable for sensing amplification to some extent, yet it remains unclear and interesting to compare the performance of the different strategies.

Gel electrophoresis was used in this study as an example for visualizing the interactions of the various tested salts and molecules with DNA. It is a commonly used, simple, economic biochemical technique, available in most biological, biochemical, and/or biophysical laboratories [95,96,97]. Advantages of using gel electrophoresis to read out the signals of DNA interactions with other molecules amplified by the bent DNA bows include the accessibility, simplicity, economy and broad range [96,97], which are expected to make the bent DNA bows broadly useful. On the other hand, we should emphasize that the key role of our bent DNA bows is to amplify the interactions and thus improve the sensitivity of the original detection methods and techniques in general; therefore, many existing techniques are expected to work well with the bent DNA molecules for measuring and visualizing the signals of DNA interactions with other molecules. It would be interesting to combine the bent DNA bows with other detection methods, including techniques based on fluorescence, melting temperature, calorimetry, circular dichroism, Raman spectroscopy, and nuclear magnetic resonance. Investigating how the bent DNA bows improve the sensitivity of these techniques will be one direction for future studies.

An advantage of our bent DNA bows is that, for a given length of the double-stranded segment of the DNA bow, we can vary the length of the single-stranded part to modulate the degree of bending (i.e., bending strain) of the double-stranded segment. It would be interesting to further study how the property and performance of the bent DNA amplifiers depend on bending strain. In addition, it would be worthwhile to visualize the bent DNA bows with different bending strains in the presence of different salts/molecules at different concentrations using TEM (Figure 2). Furthermore, we expect that this advantage makes the bent DNA bows useful for understanding how protein-DNA interactions depend on the curvature (or bending) of the DNA [98,99,100,101], which can be controlled in our bent DNA bows.

Lastly, the relaxed species (dimers, trimers and higher-order oligomers) from the bent DNA bows, are also interesting molecules worthwhile studying. Interesting questions include whether and how the relaxed species form secondary or tertiary structures, and where the interacting ions and molecules reside. Advanced single-molecule fluorescence techniques, such as single-molecule fluorescence resonance energy transfer (FRET), are expected to advance such understanding, as shown by the beautiful, pioneering work by several groups in this direction [83,102,103,104,105,106].

## 5. Patents

A patent (pending) was resulted from the work reported in this manuscript.

## Figures and Tables

**Table 1:** Salts/molecules and their concentrations used in this study.

Salt/Molecule	Concentrations
MgCl_2_	0, 1, 2, 3, 4, 5, 6, 7 mM
MgSO_4_	0, 1, 2, 3, 4, 5, 6, 7 mM
KCl	0, 1, 2, 3, 4, 5, 6, 7 mM
CaCl_2_	0, 1, 2, 3, 4, 5, 6, 7 mM
Al(NO_3_)_3_	0, 16, 32, 48, 64, 80, 96, 112 µM
Zn(NO_3_)_2_	0, 30, 60, 90, 120, 150, 180, 210 µM
AgNO_3_	0, 10, 20, 30, 40, 50, 60, 70, 80, 90 µM
Guanidine	0, 1, 2, 3, 4, 5, 6, 7 mM
Putrescine	0, 0.5, 1, 2, 4, 8, 16, 32 mM
Spermidine	0, 6, 12, 18, 24, 30, 36, 42 µM
Ganciclovir	0, 1, 2, 3, 4, 5, 6, 7 mM
Thiamine	0, 36, 72, 108, 144, 180, 300, 600 µM
Ethidium Bromide	0, 381, 400, 419, 438, 457, 476, 495 µM
SYBR Safe *	0, 25, 50, 60, 70, 80, 90, 100 µM

* It is assumed that the concentration of 1X SYBR Safe (commercially available from Thermo Fisher Scientific, Waltham, MA, USA) is 1 µM, according to the corresponding patent [45].

## Supplementary Materials

The following are available online at https://www.mdpi.com/1424-8220/20/11/3112/s1. **Figure S1**: Examples of full-length gels for DNA bows or linear DNA in the presence of salts at increasing concentrations: (**a**) MgCl_2_, (**b**) MgSO_4_, (**c**) KCl, (**d**) CaCl_2_, (**e**) Al(NO_3_)_3_, (**f**) Zn(NO_3_)_2_, and (**g**) AgNO_3_. Green rectangles indicate the cropping areas of the gels for the insets in Figure 3. **Figure S2****:** Examples of full-length gels for DNA bows or linear DNA in the presence of small organic molecules at increasing concentrations: (**a**) guanidine, (**b**) putrescine, (**c**) spermidine, (**d**) ethidium bromide, (**e**) SYBR safe, (**f**) ganciclovir, and (**g**) thiamine. Green rectangles indicate the cropping areas of the gels for the insets in Figure 4. **Figure S3****:** Correlation between $u^{\prime }$ (fitted from relaxed DNA) and u (fitted from bent DNA bows). Error bars represent fitting errors. The blue dashed line indicates $u^{\prime }=u$.
