# Supplementary figures and images for: Bent DNA Bows as Sensing Amplifiers for Detecting DNA-Interacting Salts and Molecules
Source: Sensors (Basel). 2020 May 31;20(11):3112. doi: 10.3390/s20113112 (PMC7309149; doi:10.3390/s20113112)

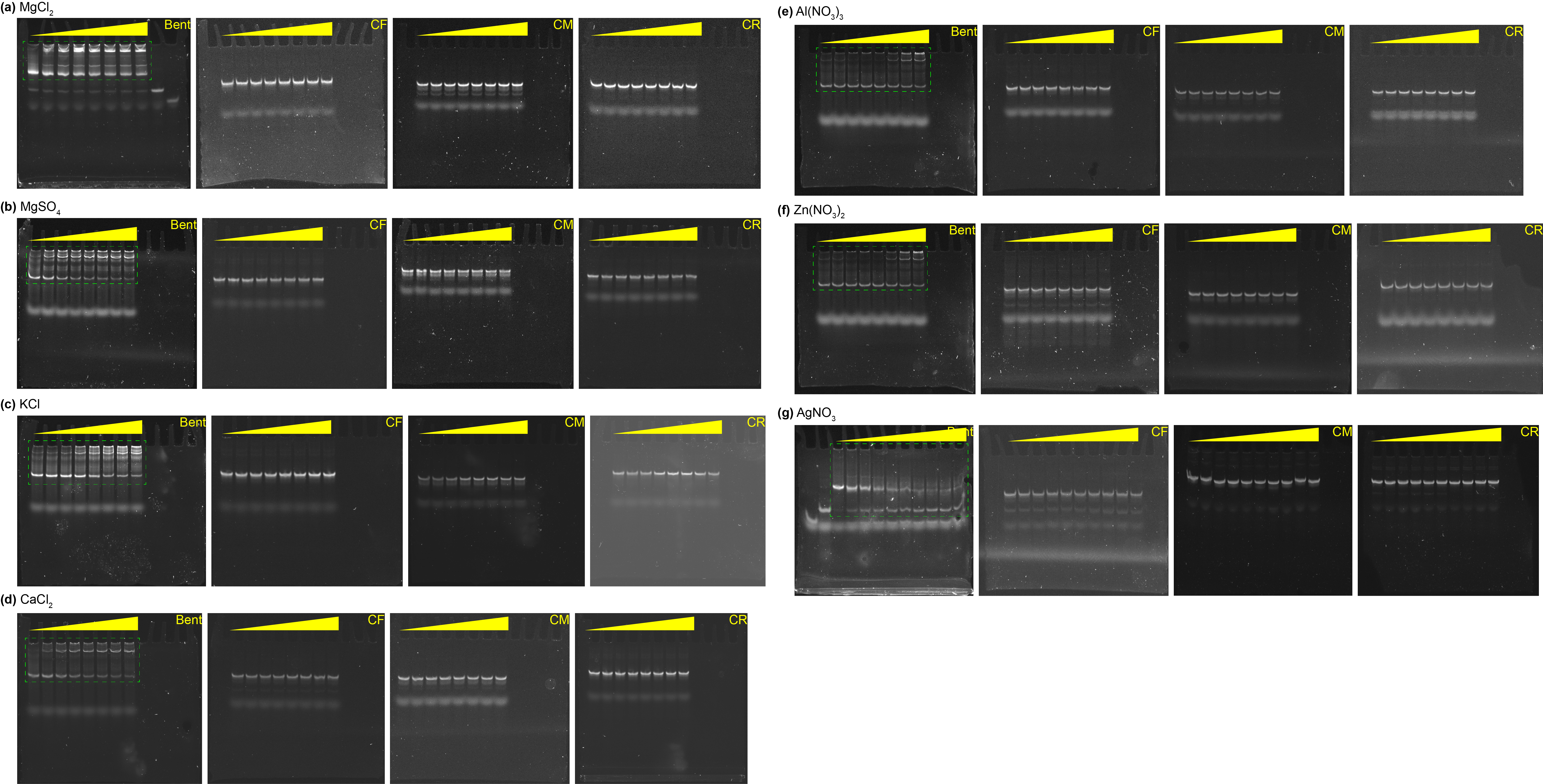

Supplement: Supplementary file 1 [file sensors-20-03112-s001.zip › FigureS1.png]

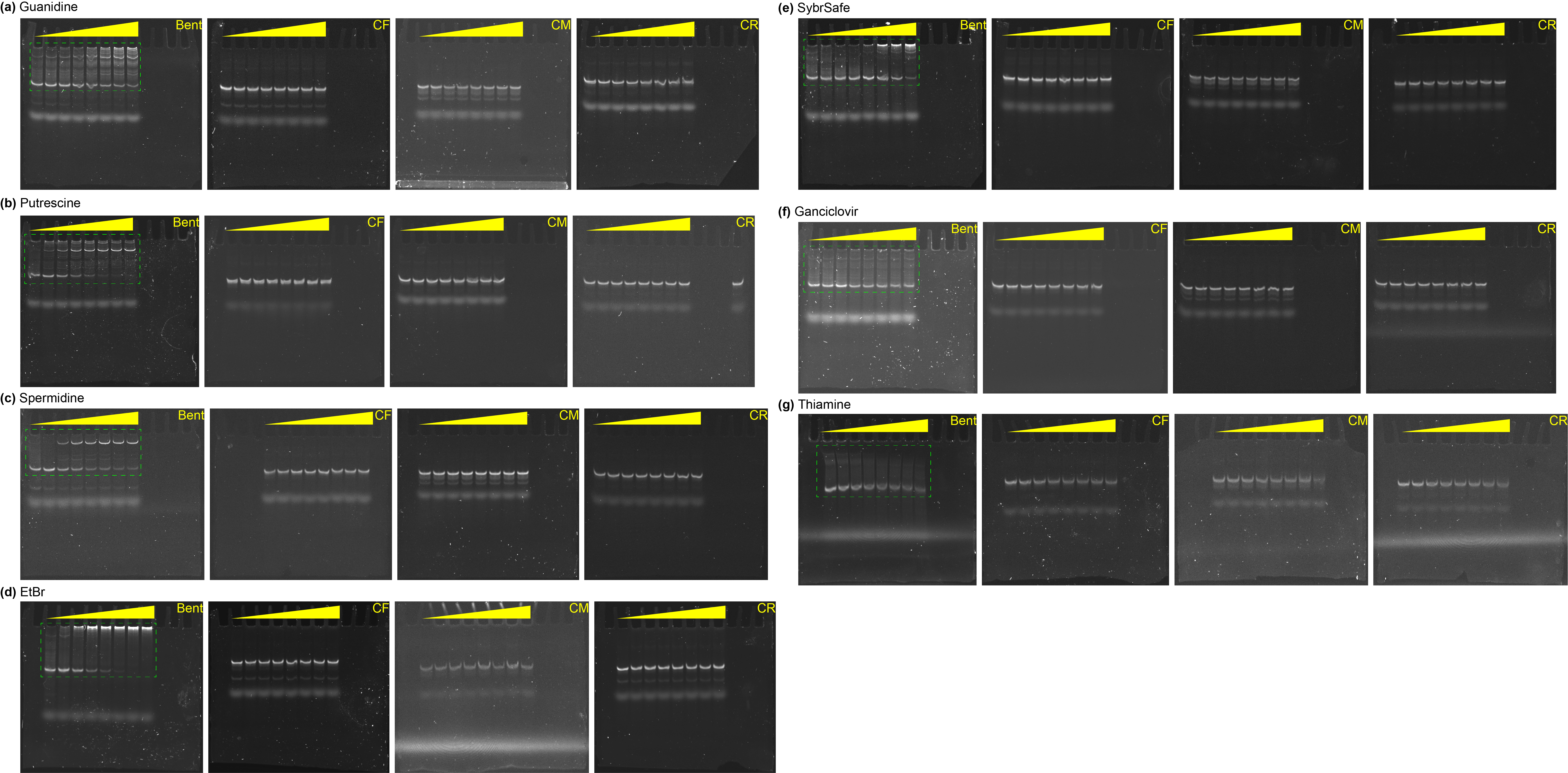

Supplement: Supplementary file 1 [file sensors-20-03112-s001.zip › FigureS2.png]

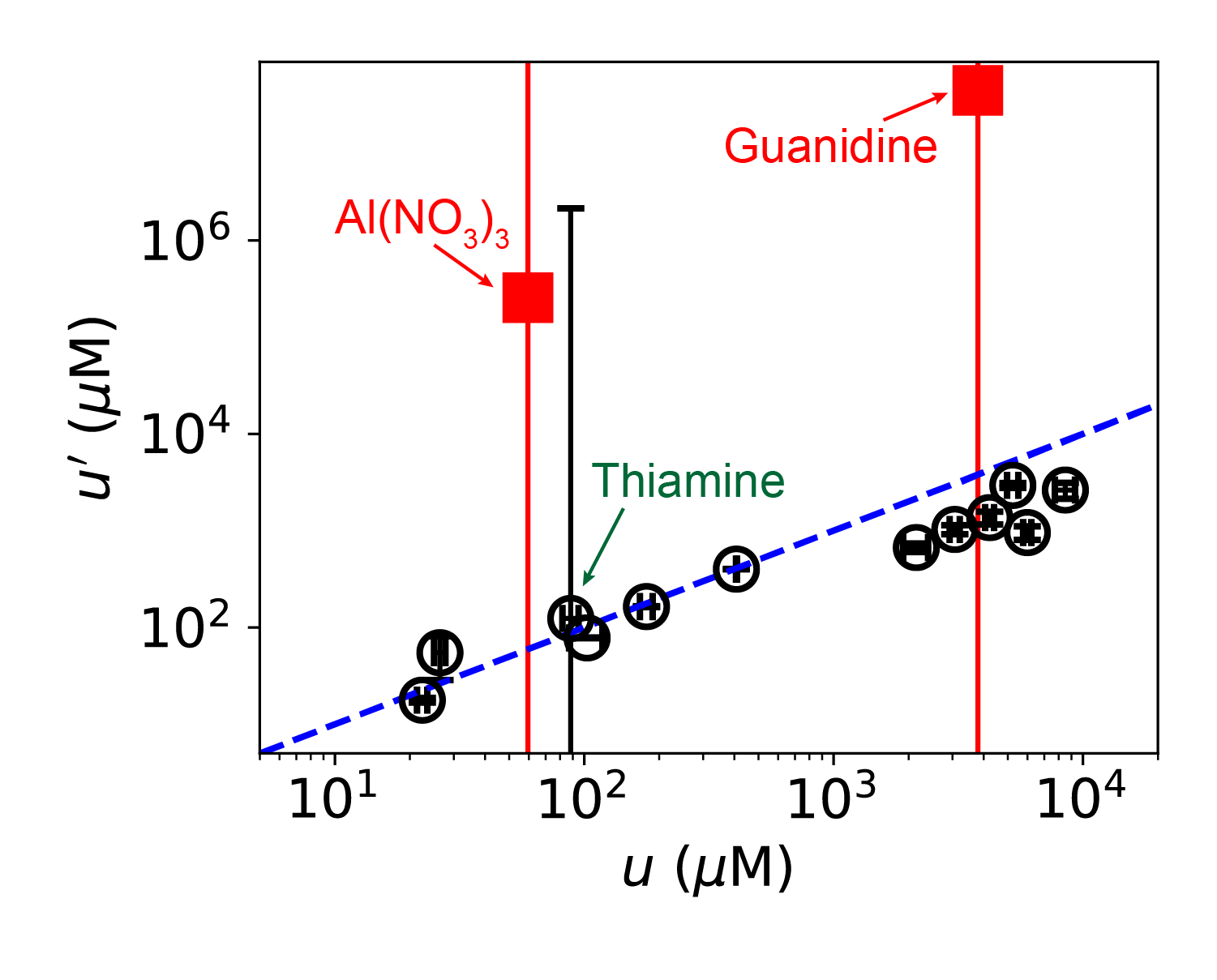

Supplement: Supplementary file 1 [file sensors-20-03112-s001.zip › FigureS3.png]
